# Molecular Mechanisms of the Anticancer Activity of Hinokitiol in C32 Melanoma Cells: Transcriptomic Analysis and Modulation by a Static Magnetic Field

**DOI:** 10.3390/molecules31203688

**Published:** 2026-10-15

**Authors:** Agnieszka Synowiec-Wojtarowicz, Celina Kruszniewska-Rajs, Agata Krawczyk, Magdalena Kimsa-Dudek

**Affiliations:** 1 Department of Nutrigenomics and Bromatology, Faculty of Pharmaceutical Sciences in Sosnowiec, Medical University of Silesia, Katowice, 8 Jednosci Street, 41-200 Sosnowiec, Poland; akrawczyk@sum.edu.pl (A.K.); mkimsa@sum.edu.pl (M.K.-D.); 2 Department of Molecular Biology, Faculty of Pharmaceutical Sciences in Sosnowiec, Medical University of Silesia, Katowice, 8 Jednosci Street, 41-200 Sosnowiec, Poland; ckruszniewska@sum.edu.pl

**Keywords:** natural products, cancer therapy, molecular mechanism, anticancer agents, bioactive compounds

## Abstract

Many studies confirm that natural substances derived from plant materials, at appropriate concentrations, can exhibit anticancer effects against various types of cancer cells. One such substance is hinokitiol, which has been proven to have anticancer effects against malignant melanoma. However, the molecular mechanisms of this effect are unknown. Therefore, the aim of this study was to determine the probable molecular mechanism of the anticancer effect of hinokitiol through gene expression analysis. Additionally, it was examined whether a ubiquitous static magnetic field could modify the anticancer properties of hinokitiol. Transcriptome analysis was performed using oligonucleotide microarrays, followed by validation of the obtained results for five genes using RT-qPCR. Fifty-six statistically different transcriptomes were found in melanoma cultures treated with only hinokitiol (*p* < 0.05, FC > 1.5). To confirm the results obtained from the microarray, RT-qPCR was performed for five genes: the *ARNTL*, *CRYAB*, *MAT2A*, *HSPA8* and *NR1D1* genes. In summary, our studies indicate that the addition of hinokitiol to melanoma cells changes the expression of numerous genes, including those associated with the biological clock. The presented results are preliminary and point out a direction for future research. The presence of a static magnetic field does not cause statistically significant changes in gene expression. Only in ARNTL was a statistically significant decrease in expression levels observed in the H+SMF group compared to the H group.

## 1. Introduction

Scientists are looking for answers to the question of how to use long-known natural substances with anticancer properties to increase the effectiveness of anticancer therapies, especially in the treatment of cancers characterized by low therapeutic potential, a high degree of malignancy and high mortality. Malignant melanoma, which can grow rapidly and metastasize, is one of the most dangerous skin cancers. As in other countries, Poland has seen an increase in cases over the past two decades, averaging 3% annually. This is likely due to increased public awareness and improved detection and reporting. Melanoma grows at a very rapid rate, especially in advanced stages, which is why the mortality rate for this cancer is approximately 40% [1]. The treatment of malignant melanoma is fraught with many difficulties, primarily related to the very rapid rate of metastasis and the high resistance of melanoma to external factors, which unfortunately leads to a significant reduction in the effectiveness of pharmacotherapy [2,3]. Therefore, scientists are exploring new possibilities for using long-known bioactive substances. Bioactive substances derived from plants are typically characterized by low toxicity, making them a particularly recommended dietary component that protects against many diseases. Many of them exhibit antioxidant properties, protecting the body against the harmful effects of free radicals, which are an etiopathological factor of many lifestyle diseases and cancers [4]. Antioxidants have increased in popularity in recent years due to the promotion of a healthy diet and their pharmacological properties. The use of antioxidants as an alternative combined anticancer therapy is the subject of much research. One such substance with antioxidant properties is Thujaplicin, also known as hinokitiol. Hinokitiol exhibits health-promoting and pharmacological effects, including antioxidant, anti-inflammatory, antifungal, and anticancer properties [5,6,7]. Hinokitiol has proven anticancer properties while also exhibiting protective effects on normal cells. Studies confirm that hinokitiol can lead to the death of various types of cancer cells through apoptosis and may also inhibit the process of metastasis and increase therapeutic success [8]. Hinokitiol has also demonstrated significant antitumor activity in many cancers derived from different cell types, including melanoma, lung cancer, endometrial cancer, breast cancer, and rare cancers such as osteoma and adenocarcinoma. In a study by Chiang et al., hinokitiol at a concentration of 50µM demonstrated potent anticancer activity against breast cancer (BC) cells by initiating cell apoptosis and increasing p-53 protein expression [9]. On the other hand, Seno et al. demonstrated that hinokitiol at concentrations above 20 µM could cause apoptosis in lung cancer cells (NCI-H460) by inhibiting and degrading XIAP [10]. In our previous studies, we confirmed that 40 µM hinokitiol exhibits anticancer activity against malignant melanoma cells (C32 and COLO 829) by disturbing cell redox homeostasis [11]. The anticancer effects of bioactive ingredients can be modified by many factors, some of which work synergistically with natural substances, enhancing these properties. However, a number of factors can decrease this anticancer effect and act as a “protector” for cancer cells. One such factor may be a static magnetic field, which accompanies humans in everyday life as it is emitted by household appliances, industrial equipment, and the increasingly popular MRI scanners used to diagnose various diseases, including cancer, and to monitor the effects of cancer pharmacotherapy [12]. In Poland, MRI scanners most commonly use a magnetic field in the range of 0.2 to 3T, while in some EU countries, MRI with magnetic fields as high as 8 T is used. During examinations, patients and staff are exposed to a magnetic field, which may have health consequences for both the patients and the medical staff. Contrast agents are often used during MRI examinations, administered when the scanner’s electromagnetic field is turned off. However, it is not possible to stop permanent magnets from generating a static field [13,14]. Scientists have been trying for years to answer the question of whether a static magnetic field has a positive or negative effect on the human body. There are numerous publications whose primary research goal was to explain the field’s effects on cell cultures, laboratory animals, and humans, and numerous studies suggest that it may impair the proper functioning of cells or the entire organism. The research results available in the literature are inconsistent because studies are conducted on various cell types, including normal and cancer cells, and varying induction of SMF and different sources of SMF are used in the studies [15,16]. There are many scientific studies claiming that a static magnetic field can affect cell orientation, proliferation, gene expression, cell metabolic activity or cell membrane structure [17]. Some studies also confirm that a static magnetic field may have anticancer effects. One of the suggested mechanisms of SMF anticancer activity involves the induction of oxidative stress in cancer cells, which may lead to apoptosis of these cells. On the other hand, many researchers suggest that increased exposure to a static field may lead to a disturbance of the oxidation–reduction of normal cells, leading to their apoptosis [18,19].

Due to increasing resistance to chemotherapy, its harmful effects on healthy tissues and cells, and the frequent recurrence of cancer, scientists are constantly looking for alternative methods of fighting cancer. The aim of our research was to understand the molecular mechanism of hinokitiol against malignant melanoma cells by analyzing gene expression using oligonucleotide microarrays and RT-qPCR. Additionally, we tested whether a static magnetic field would modulate the anticancer properties of hinokitiol.

## 2. Results

**Figure 1:**
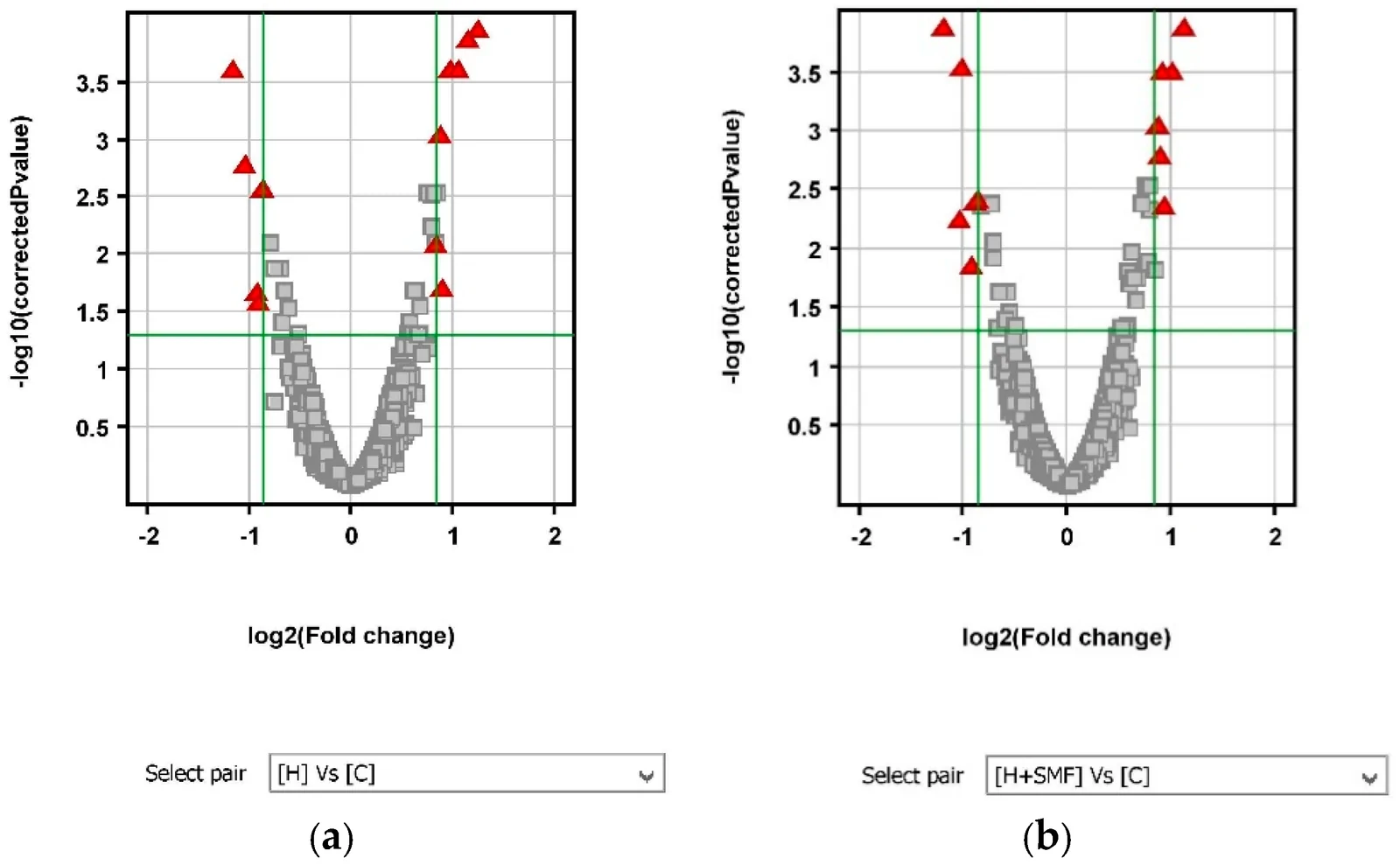
Volcano plots of gene expression differences in malignant melanoma cells: (**a**) cells treated with hinokitiol vs. control cells; (**b**) cells simultaneously exposed to hinokitiol and placed in test chamber with SMF vs. control cells. (red triangles—statistically significant genes, gray squares—statistically insignificant genes).

Four groups of C32 melanoma cell cultures—control cells (C), cells treated with hinokitiol (40 µM) (H), cells placed in SMF-emitting test chambers (0.7T) (SMF), and cells treated with hinokitiol and put into SMF-emitting permanent magnets (H+SMF) (Figure 1, Table 1)—were used to understand the molecular mechanism of hinokitiol’s action on C32 cells.

**Figure 2:**
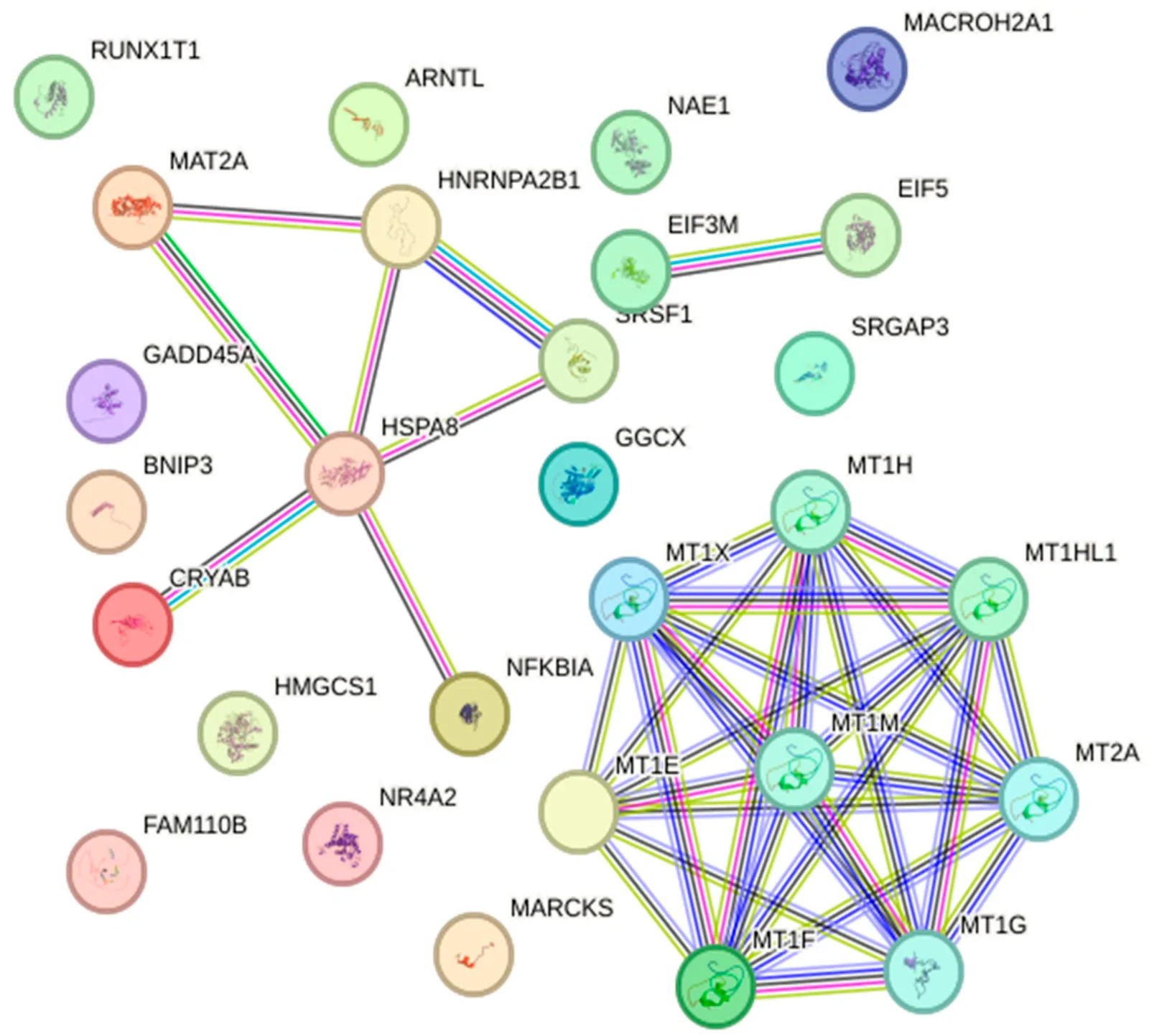
Relationships between statistically significant genes in malignant melanoma cells (STRING database version 12.5).

Comparing the transcriptome results of C32 cells cultured with hinokitiol (H) and control cells without any additives (C) identified 56 significantly changed gene expressions (*p* < 0.05, FC > 1.5). Among these transcripts, 36 were upregulated, while 20 were downregulated. In turn, in the melanoma cells that had been simultaneously exposed to hinokitiol and a static magnetic field (H+SMF), 47 statistically significant differentially expressed transcripts were detected compared to the control cells (*p* < 0.05, FC > 1.5). Among these transcripts, 25 statistically significant differentially expressed genes were upregulated and 22 were downregulated (Table 2). Figure 2 shows the relationships between genes whose expression was statistically significant (*p* < 0.05, FC > 1.5) in cells simultaneously exposed to hinokitiol 40 µM and put in a magnetic chamber with SMF 0.7T in relation to the control melanoma cells (C32).

**Figure 3:**
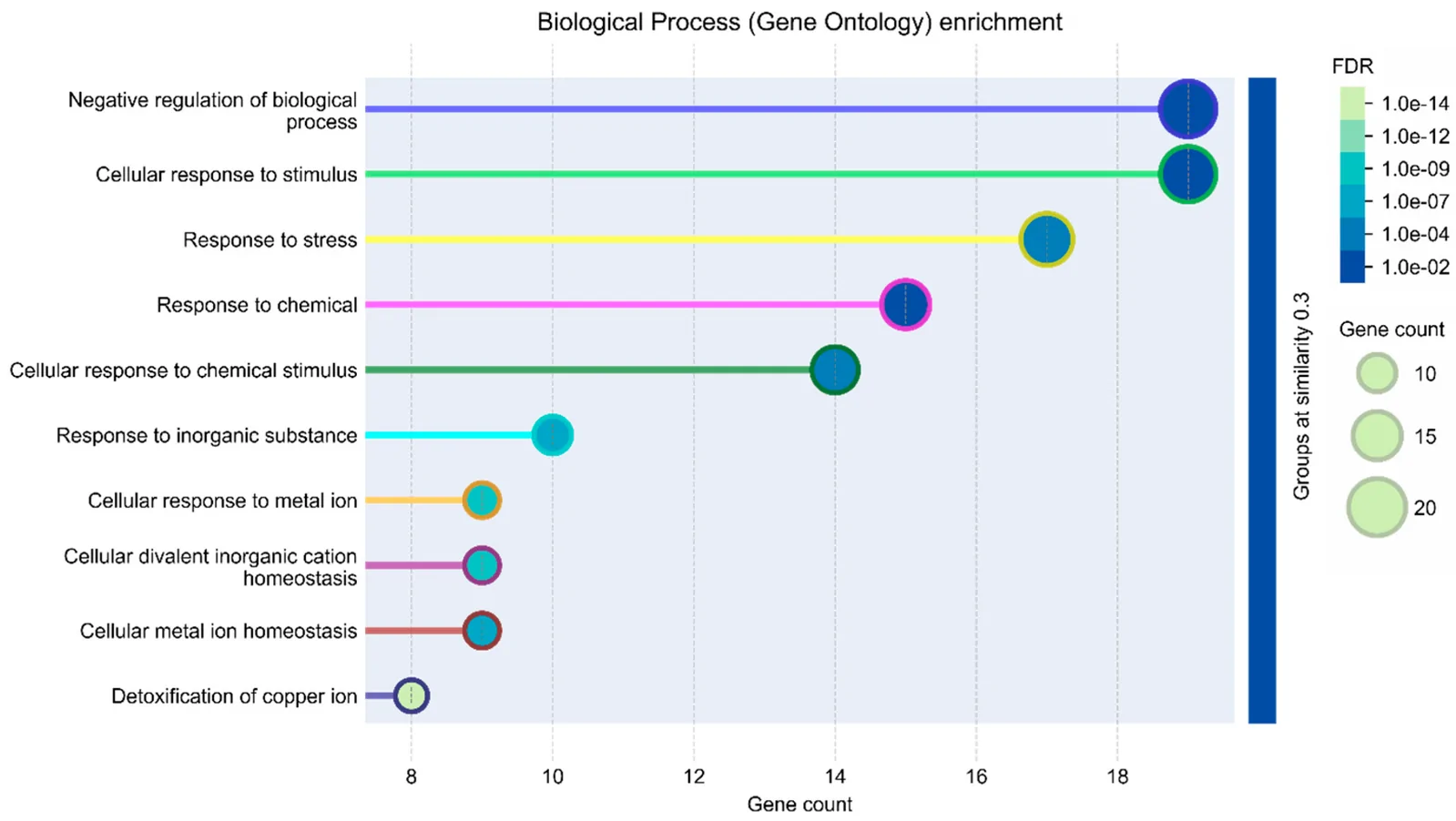
Differentiating genes involved in cellular biological processes (STRING database, version 12.5).

STRING database analysis showed that the genes for which expression was statistically significant (*p* < 0.05 and FC > 1.5) in cells treated with hinokitiol and exposed to an SMF, in relation to the control cells, participate in the following biological processes: cellular response to stimulus (19 genes); response to stress (17 genes); response to chemical (15 genes); cellular response to chemical stimulus (14 genes); response to inorganic substance (10 genes); cellular response to metal ion, cellular divalent inorganic cation homeostasis and cellular metal ion homeostasis (nine genes each); and detoxification of copper ion (eight genes) (Figure 3).

**Figure 4:**
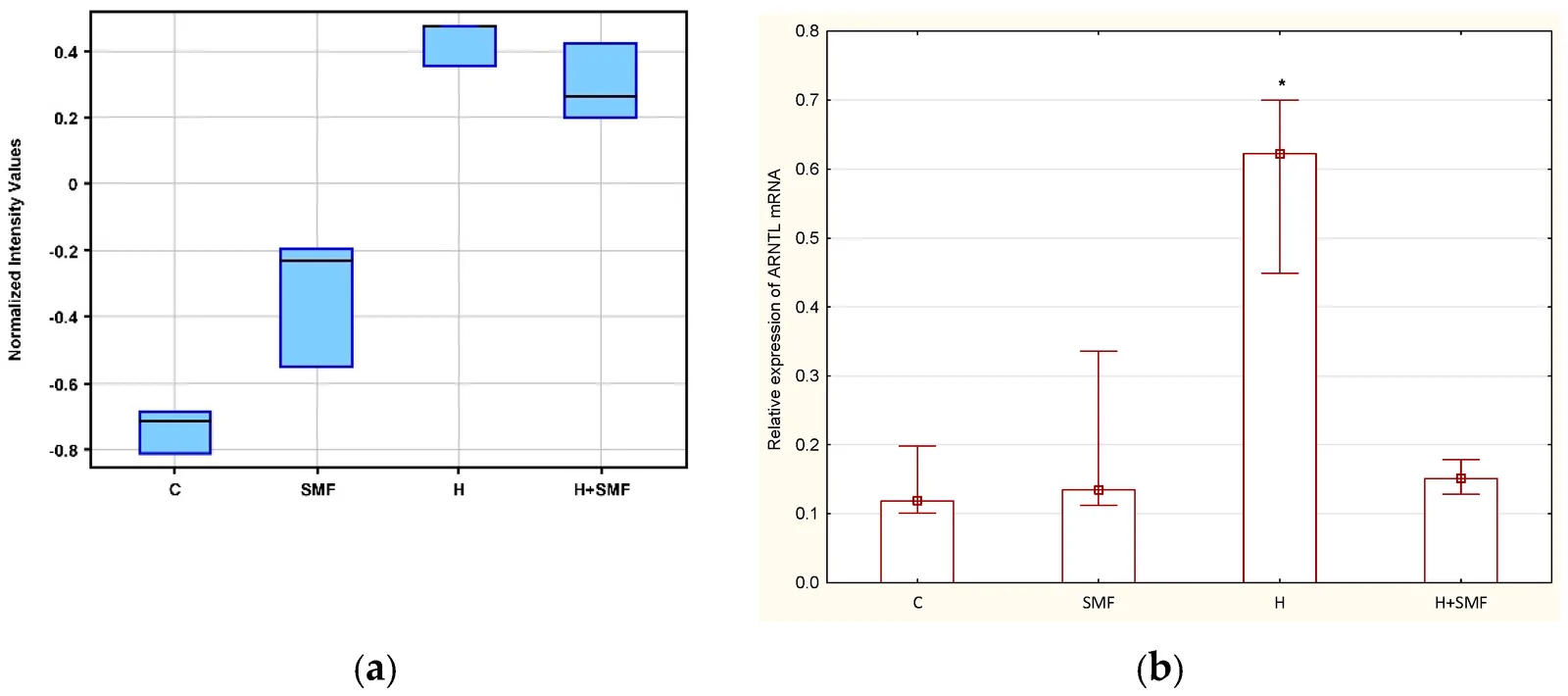
Comparison of changes in ARNTL gene expression using the oligonucleotide microarray technique (**a**) and the RT-qPCR technique (**b**) in all study groups: C—control C32, H—C32 with hinokitiol, SMF—C32 put in test chamber, H+SMF—C32 with hinokitiol and placed in magnetic test chamber. * *p* < 0.05 vs. C (control) (Kruskal–Wallis test H = 21.33, *p* < 0.001).

**Figure 5:**
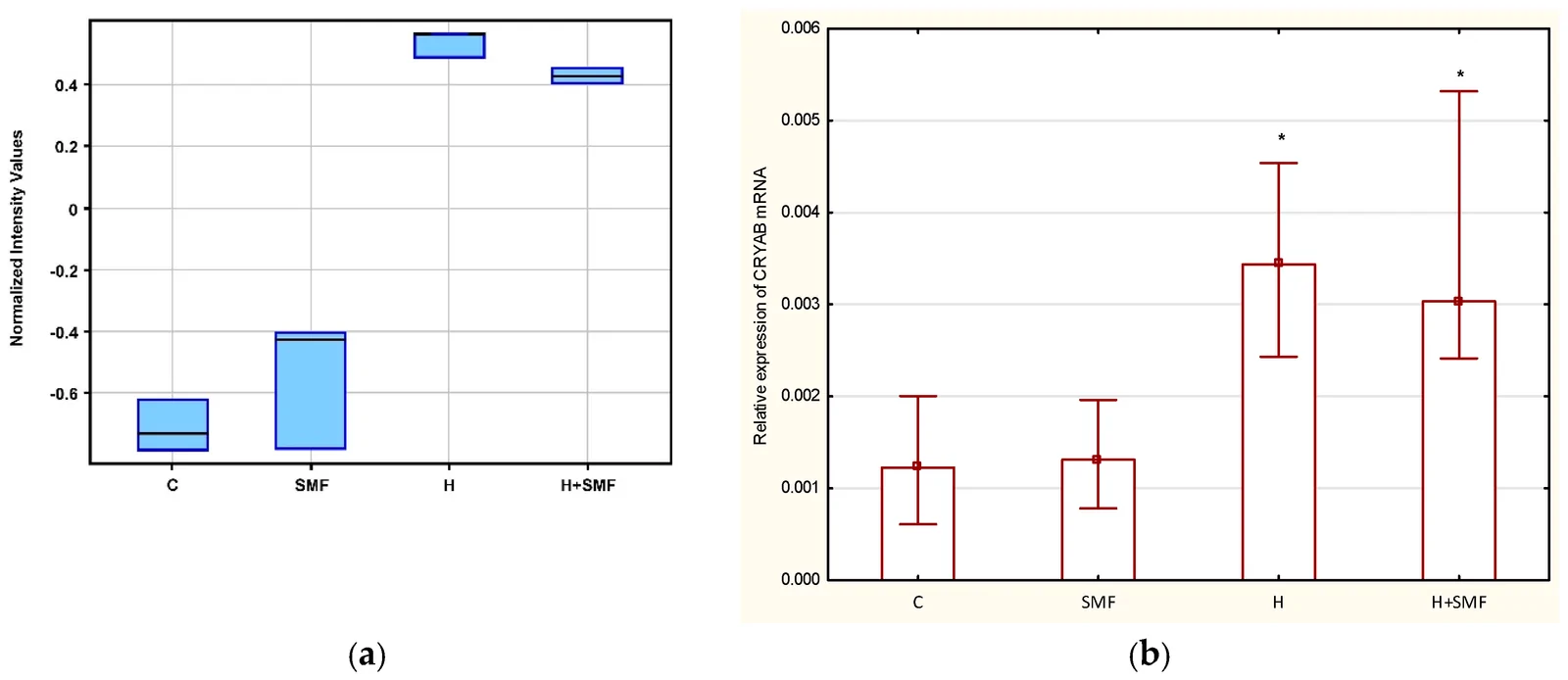
Comparison of changes in CRYAB gene expression using the oligonucleotide microarray technique (**a**) and the RT-qPCR technique (**b**) in all study groups: C—control C32, H—C32 with hinokitiol, SMF—C32 put in test chamber, H+SMF—C32 with hinokitiol and placed in magnetic test chamber. * *p* < 0.05 vs. C (control) (Kruskal–Wallis test H = 25.71, *p* < 0.001).

**Figure 6:**
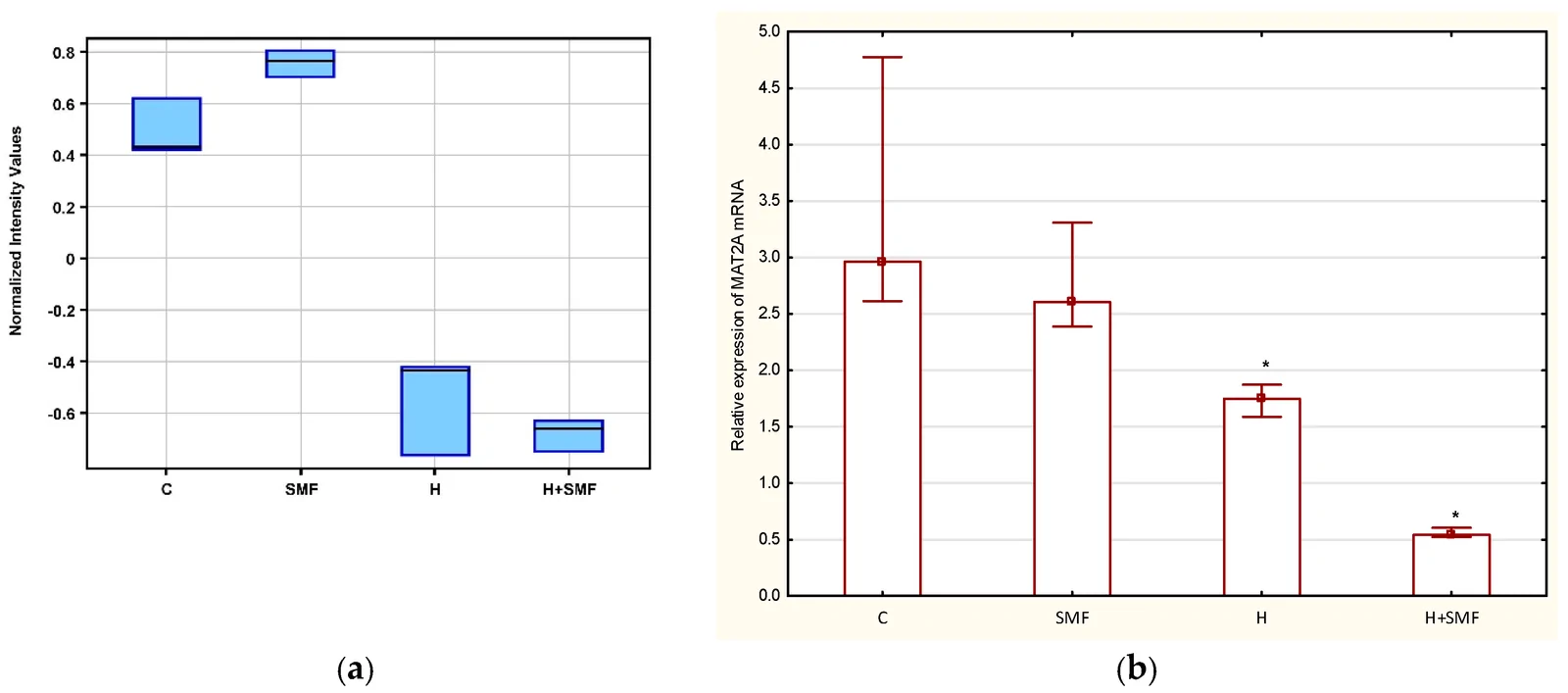
Comparison of changes in MAT2A gene expression using the oligonucleotide microarray technique (**a**) and the RT-qPCR technique (**b**) in all study groups: C—control C32, H—C32 with hinokitiol, SMF—C32 put in test chamber, H+SMF—C32 with hinokitiol and placed in magnetic test chamber. * *p* < 0.05 vs. C (control) (Kruskal–Wallis test H = 28.50, *p* < 0.001).

**Figure 7:**
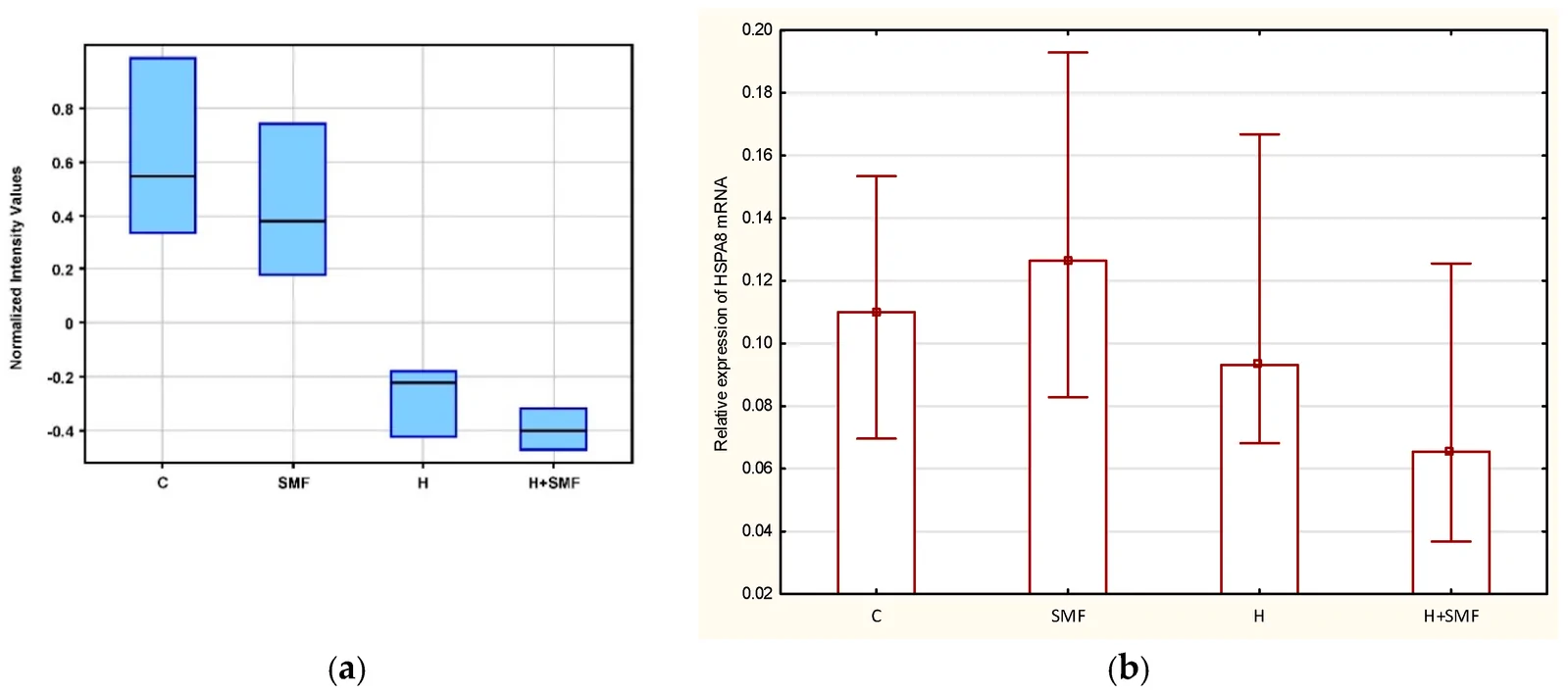
Comparison of changes in HSPA8 gene expression using the oligonucleotide microarray technique (**a**) and the RT-qPCR technique (**b**) in all study groups: C—control C32, H—C32 with hinokitiol, SMF—C32 put in test chamber, H+SMF—C32 with hinokitiol and placed in magnetic test chamber. (Kruskal–Wallis test H = 8.64, *p* = 0.034.)

**Figure 8:**
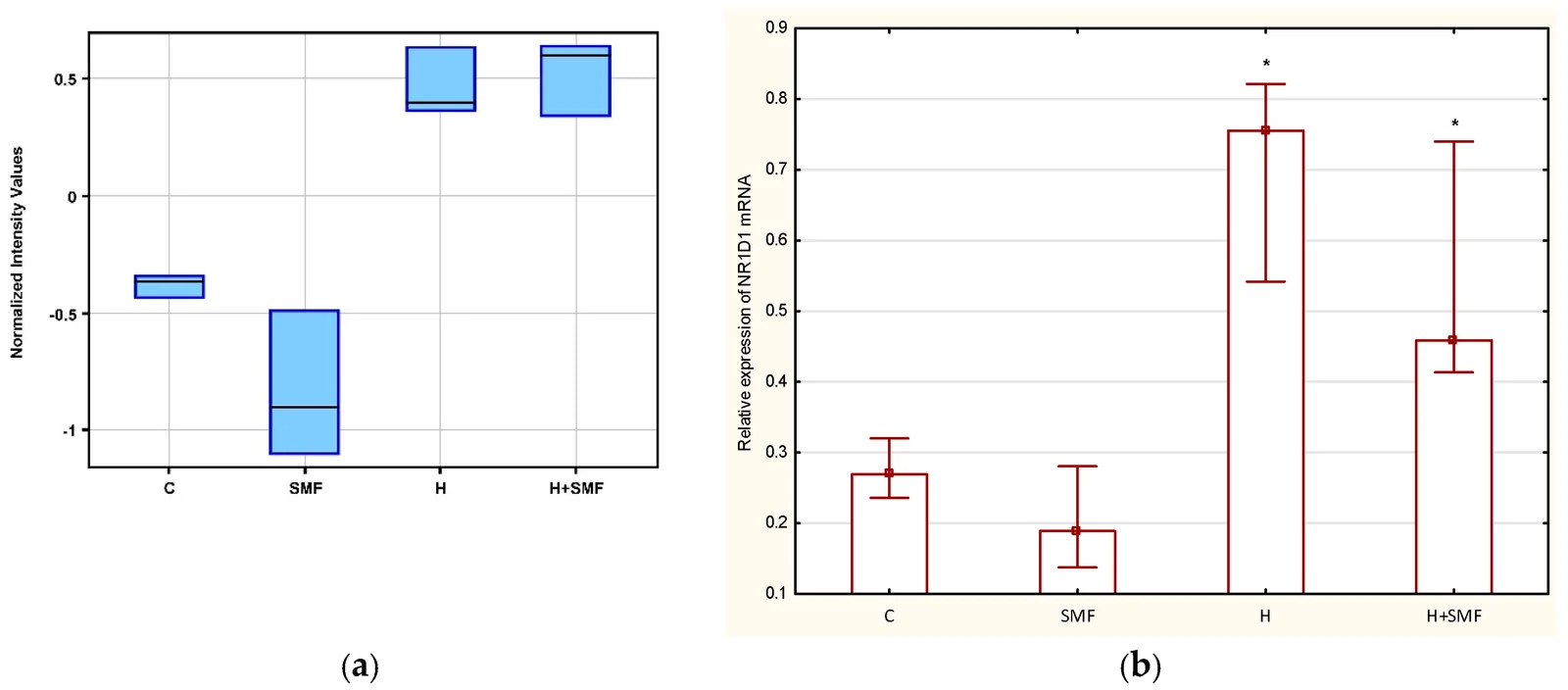
Comparison of changes in NR1D1 gene expression using the oligonucleotide microarray technique (**a**) and the RT-qPCR technique (**b**) in all study groups: C—control C32, H—C32 with hinokitiol, SMF—C32 put in test chamber, H+SMF—C32 with hinokitiol and placed in magnetic test chamber. * *p* < 0.05 vs. C (control) (Kruskal–Wallis test H = 30.07, *p* < 0.001).

To validate the results obtained using the RT-qPCR technique, five genes were selected for which the highest FC value was determined (FC ≥ 1.70): *ARNTL*, *CRYAB*, *MAT2A*, *HSPA8* and *NR1D1*. Analysis of the obtained RT-qPCR confirmed the previous results obtained using oligonucleotide microarrays in C32 cultures supplemented with hinokitiol and placed in SMF-emitting test chambers (Figure 4, Figure 5, Figure 6, Figure 7 and Figure 8).

To determine whether an SMF modifies the effect of hinokitiol on gene expression, a two-way nonparametric analysis of variance (Scheirer–Ray–Hare test) was performed. The analysis showed a significant effect of hinokitiol on all selected genes, *ARNTL*, *CRYAB*, *MAT2A*, *HSPA8* and *NR1D1* (*p* < 0.05), whereas the SMF had no significant main effect on any of them. A significant H x SMF interaction was found only for the *ARNTL1* gene (*p* = 0.005), indicating that an SMF modifies the action of hinokitiol: the increase in *ARNTL1* expression induced by hinokitiol was abolished in the presence of the SMF.

## 3. Discussion

Excessive sun exposure and genetic predisposition may be factors increasing the risk of malignant melanoma, the predominant cause of death from skin cancer. The earlier the disease is diagnosed, the better the prognosis for patients, as this type of cancer metastasizes very quickly and has a high tendency to undergo malignant transformation [20]. Melanoma causes nearly 75% of deaths from skin cancer, and 92 000 new cases of melanoma are reported worldwide annually. Despite many studies, the etiopathogenesis of melanoma has not yet been fully elucidated. Malignant melanoma develops in the skin in 90% of cases. It can also develop in the eyeball, meninges, or mucous membranes. It can arise in: pigmented nevi, primarily hairless and particularly in congenital giant nevi, malignant lentigines and junction nevi. Melanoma can arise de novo from otherwise intact skin. A large percentage of melanomas arise from atypical moles and atypical mole syndrome [21].

Due to the increasing resistance to chemotherapy, its harmful effects on healthy tissues and cells, and the frequent recurrence of cancer, scientists are constantly looking for alternative methods of fighting cancer. The aim of our study was to investigate which genes show expression changes in malignant melanoma cells treated with hinokitiol (40 µM) and put into a magnetic chamber with an SMF of 0.7 T. To achieve this aim, oligonucleotide microarrays were used. Our previous studies confirmed that hinokitiol exhibits anticancer effects on malignant melanoma cells. One of the mechanisms of its anticancer action is disrupting oxidation–reduction homeostasis by reducing the activity of antioxidant enzymes. Furthermore, a static magnetic field demonstrated a protective effect on melanoma cells and abolished the anticancer effects of hinokitiol [11]. In this study, 22,277 mRNAs were analyzed in control melanoma cells, cells exposed to SMF, cells treated with hinokitiol, and cells simultaneously exposed to hinokitiol and a static magnetic field. We found statistically significant differences in 56 gene transcripts, primarily involved in cellular response to stimulus, stress response, and response to chemicals. To validate the obtained results, selected genes with FC greater than 1.7 (ARNTL, CRYAB, MAT2A, HSPA8 and NR1D1) were analyzed using RT-qPCR.

Aryl hydrocarbon receptor nuclear translocator-like—ARNTL—is a transcriptional activator that forms a core component of the circadian clock. One of the newest types of cellular autophagy, described in 2019, is clockophagy, which involves the autophagic removal of one of the most important proteins of the biological clock, ARNTL. Clockophagy has been shown to be involved in ferroptosis, for example, in pancreatitis. Therefore, understanding the mechanisms of clockophagy in the ferroptosis of cancer cells dependent on ARNTL pathways may represent a potential therapeutic avenue for ferroptosis-based anticancer treatment [22,23]. In our study, we found that the addition of 40 µM of hinokitiol to malignant melanoma cell cultures caused a statistically significant increase in ARNTL expression. In a study by Kong et al., high ARNTL expression positively correlated with prognosis and long survival in patients with osteosarcoma [24]. Analyzing pancreatic cancer cells’ resistance to gemcitabine, Jiang et al. also found that this mechanism is related to ARNTL and its low expression in tumor cells [25]. Zeng et al. proved that ARNTL may be a good marker in colorectal cancer [26]. Another analyzed gene whose expression was statistically higher in cultures treated with hinokitiol and those treated with hinokitiol and an SMF was NR1D1, which, like ARNTL, is a gene controlling the biological clock. Zhu et al. analyzed the expression of biological clock genes in healthy tissue and ovarian cancer samples and found low expression in cancer tissues of the ARNTL and NR1D1 genes, similarly to our study; in control melanoma cells the expression of both ARNTL and NR1D1 was low compared to that in cultures treated with hinokitiol [27]. The next analyzed gene that was upregulated (FC = 2.45—H vs. C; FC = 2.23—H+SMF vs. C) was CRYAB, also known as HspB5 or αB-crystallin, which is a heat shock protein implicated in numerous diseases, including heart and eye diseases, and some cancers (ovarian, breast, and prostate cancer) [28]. Similarly, Qiao et al. confirmed that the addition of Thiostrepton to melanoma cell cultures demonstrated antitumor activity through upregulation of heat shock genes, including CRYAB [29]. On the other hand, Tan et al.’s study demonstrated that high CRYAB and p53 expression increases the risk of metastasis in ovarian cancer patients [30]. Jayakumar et al., analyzing chaperones in patients with cholangiocarcinoma, concluded that CRYAB may be a good therapeutic marker, and its expression can be modulated by the antichaperone drugs tucatinib and regorafenib [31]. Methionine adenosyltransferase 2A (MAT2A), which encodes an enzyme that catalyzes the formation of S-adenosylmethionine from methionine and ATP, is a protein that is deregulated in many cancers. This study also found that MAT2A showed downregulation in cells treated with hinokitiol or co-exposed to hinokitiol and an SMF. Furthermore, studies by Zhang et al. confirmed that norcantharidin exerts anticancer activity in ovarian cancer by reducing S-adenosylmethionine levels and inhibiting the MAT2A-dependent signaling pathway [32]. Also, Li et al. demonstrated that the synthetic compound 2(1H)-Quinoxalinone exhibits antitumor activity against murine melanoma cells via MAT2A inhibition [33].

The diversity of chemical structures and pharmacological activities of natural substances provides a starting point for the discovery of new anticancer drugs and understanding their molecular mechanisms of action. One such substance with documented anticancer activity is hinokitiol, which also exhibits a broad spectrum of activity against viruses, bacteria, and fungi. Recent scientific reports have confirmed that hinokitiol affects iron metabolism in cells and may protect against oxidative stress and influence cell ferroptosis by inhibiting JNK signaling pathways [34,35]. An anticancer effect on malignant melanoma cells—mediated by the induction of ferroptosis—was also confirmed in a study by Zhang et al., who investigated salidroside, a compound derived from *Rhodiola rosea* [36]. Studies by other researchers have also shown that natural compounds can exhibit anticancer activity against malignant melanoma cells. In studies by Liu et al., Honokiol at a concentration of 10–50μM was found to have a cytotoxic effect on malignant melanoma cells, the authors citing binding to the KRT18 protein as its mechanism of action [37]. In turn, in a study by Hu et al., it was found that 11 compounds derived from Morus alba L showed IC50 values from 5μM to 30μM against malignant melanoma cells. For Sanggenon C, the molecular mechanism was confirmed to involve the disruption of intracellular Ca^2+^ concentration homeostasis, leading to melanoma cell apoptosis [38].

In our study, only two of the investigated genes—ARNTL and NR1D1—show potential associations with clockophagy. However, these results are preliminary and require further targeted research and validation through the analysis of other signaling pathways and proteins, as well as measurements of LC3-I/LC3-II and p62 levels, assessments of autophagic flux, and functional inhibition of autophagy. The remaining genes studied—CRYAB, HSPA8, and MAT2A—play roles in stress response, protein homeostasis, metabolism, and cell growth. Furthermore, our results indicate that an SMF only modifies the action of hinokitiol regarding ARNTL gene expression, suggesting a tendency to normalize the changes induced by this compound in the expression of the ARNTL gene, which is associated with clockophagy.

## 4. Materials and Methods

In this study, a malignant melanoma cell line (C32) (ATCC CRL-1585, Manassas, VA, USA) was cultured with hinokitiol and placed in magnetic chambers emitting an SMF.

### 4.1. Chemicals

All reagents and media used were purchased from: Invitrogen, Carlsbad, CA, USA; Lonza, Basel, Switzerland; and Sigma-Aldrich, St. Louis, MO, USA.

### 4.2. Cell Culture

The conditions for culturing melanoma cells were described in an earlier work. This earlier study also presented the results of a cytotoxicity assessment of hinokitiol 5–80 µM on melanoma cells and normal melanocytes using the MTT (Sigma-Aldrich, St. Louis, MO, USA)viability assay [11]. For this experiment, a hinokitiol (Sigma-Aldrich, St. Louis, MO, USA) concentration of 40 µM was chosen because it was not toxic to melanocytes but was cytotoxic to C32 cells. The tested cells were then treated with hinokitiol and placed in magnetic chambers emitting an SMF of 0.7 T. The culture conditions in the static magnetic field and the magnetic chamber were described in previous studies [39]. Then, after 24 h of treatment with hinokitiol and the static magnetic field, C32 cells were harvested for further study steps.

### 4.3. Transcriptional Activity in Melanoma Cells

TRIzol (Invitrogen, Carlsbad, CA, USA) reagent was used for total RNA extraction, and isolation was performed according to the manufacturer’s instructions. Quantitative and qualitative assessment of the obtained RNA extracts was also performed, as described in previous studies [40]. The transcriptional activity of genes in melanoma cells treated with hinokitiol and placed in magnetic chambers emitting an SMF, and that in control cells without chemical or physical exposure, or exposed only to hinokitiol or SMF, was determined using the microarray technique (HG-U133A 2.0, Affymetrix, Santa Clara, CA, USA) according to the manufacturer’s protocols. Twelve cultures were used to evaluate transcriptional activity using oligonucleotide microarrays: 3 control cultures of C32 cells (C), three cultures of C32 cells cultured with the addition of hinokitiol 40 µM (H), three cultures in which C32 cells were placed in magnets emitting an SMF with an induction of 0.7T (SMF) and 3 cultures in which C32 cells were exposed to the simultaneous action of hinokitiol and an SMF (H+SMF). The synthesis of biotinylated cRNA and its fragmentation were performed using a Gene-Chip 3′ IVT Express Kit (Affymetrix). Hybridization with the microarray and staining of hybridized cRNA with streptavidin–FITC were carried out by means of a GeneChip Hybridization, Wash, and Stain Kit (Affymetrix), according to the manufacturer’s instructions. Fluorescence intensity was measured using a Gene Chip Scanner 3000 7G and GeneChip Command Console Software (version 4.3.4) (Affymetrix). Next, to validate the results obtained at the transcriptome level, the RT-qPCR technique was used.

### 4.4. Quantitative Real-Time RT-PCR Assay

The gene expression of ARNTL (aryl hydrocarbon receptor nuclear translocator-like), CRYAB (crystallin alpha B), HSPA8 (heat shock protein family A (Hsp70) Member 8), MAT2A (methionine adenosyltransferase 2A), NR1D1 (nuclear receptor subfamily 1, group D, member 1) and ACTB (β-actin) was measured using a two-step real-time RT-qPCR SensiFAST™ cDNA Synthesis Kit and a SensiFAST™ SYBR^®^ No-ROX Kit (Bioline, London, UK), specific primers and a LightCycler^®^ 480 Instrument II (Roche Life Science, Basel, Switzerland). Reaction parameters: polymerase activation at 95 °C for 2 min; denaturation via 40 cycles of 95 °C for 5 s, annealing at 60 °C for 10 s, and extension at 72 °C for 5 s.

### 4.5. Statistical Analyses

The oligonucleotide microarray data were analyzed using the GeneSpring 13.0 platform (Agilent Technologies UK Ltd., South Queensferry, UK) and PL-Grid infrastructure. Based on the FC values, the following genes were selected for the next stage of the study: ARNTL, CRYAB, HSPA8, MAT2A and NR1D1. A list of genes whose expression, determined by the microarray technique, was greater or less than 1.5 FC was prepared. The Kruskal–Wallis test followed by post hoc multiple comparisons was used to evaluate the results of real-time RT-qPCR. To assess whether an SMF modifies the action of H, a two-way nonparametric analysis of variance (Scheirer–Ray–Hare test) was performed to evaluate the effects of H, SMF and their interaction (H × SMF). The analysis was based on ranked data, and the test statistic was compared against the χ^2^ distribution (df = 1). Analyses were conducted using STATISTICA 13.0, and statistical significance was defined at *p* < 0.05.

## Figures and Tables

**Table 1:** Numbers of probe set identifiers (IDs) of genes differentiating transcripts of melanoma cells treated with hinokitiol and exposed to static magnetic field.

One-Way ANOVA with Benjamini–Hochberg Correction					
P Complete	*p* < 0.050	*p* < 0.020	*p* < 0.010	*p* < 0.005	*p* < 0.001
22,277	56	25	20	11	2

**Table 2:** Analysis of differentially expressed transcriptomes in C32 cell cultures supplemented with hinokitiol versus controls (H vs. C) and C32 cells simultaneously exposed to hinokitiol and a static magnetic field (H+SMF) versus controls (H+SMF vs. C). FC—fold change; ↑, ↓—higher and lower expression compared to the control cells.

ID mRNA	Gene Symbol	Gene Name	FC	
			H vs. C	H+SMF vs. C
209283_at	*CRYAB*	crystallin alpha B	2.45 ↑	2.23 ↑
200768_s_at	*MAT2A*	methionine adenosyltransferase II, alpha	2.23 ↓	2.14 ↓
201502_s_at	*NFKBIA*	nuclear factor of kappa light polypeptide gene enhancer in B-cells inhibitor, alpha	2 ↑	1.87 ↑
209824_s_at	*ARNTL*	aryl hydrocarbon receptor nuclear translocator-like	2.25 ↑	1.95 ↑
217165_x_at	*MT1F*	metallothionein 1F	1.88 ↑	1.94 ↑
209794_at	*SRGAP3*	SLIT-ROBO Rho GTPase activating protein 3	1.77 ↑	1.72 ↑
213705_at	*GGCX*	gamma-glutamyl carboxylase	1.83 ↓	1.8 ↓
208581_x_at	*MT1X*	metallothionein 1X	1.7 ↑	1.7 ↑
214500_at	*H2AFY*	H2A histone family, member Y	2.04 ↑	1.69 ↑
203725_at	*GADD45A*	growth arrest and DNA-damage-inducible, alpha	1.83 ↑	1.77 ↑
204622_x_at	*NR4A2*	nuclear receptor subfamily 4, group A, member 2	1.81 ↑	1.62 ↑
221959_at	*FAM110B*	family with sequence similarity 110, member B	1.71 ↑	1.96 ↑
210338_s_at	*HSPA8*	heat shock 70kDa protein 8	1.58 ↓	1.73 ↓
201848_s_at	*BNIP3*	BCL2/adenovirus E1B 19kDa interacting protein 3	1.75 ↑	1.76 ↑
201669_s_at	*MARCKS*	myristoylated alanine-rich protein kinase C substrate	1.75 ↓	1.8 ↓
205292_s_at	*HNRNPA2B1*	heterogeneous nuclear ribonucleoprotein A2/B1	1.68 ↓	1.72 ↓
204760_s_at	*NR1D1*; *THRA*	nuclear receptor subfamily 1, group D, member 1; thyroid hormone receptor, alpha	1.68 ↑	1.96 ↑
216248_s_at	*NR4A2*	nuclear receptor subfamily 4, group A, member 2	1.76 ↑	1.71 ↑
212859_x_at	*MT1E*	metallothionein 1E	1.53 ↑	1.59 ↑
205822_s_at	*HMGCS1*	3-hydroxy-3-methylglutaryl-CoA synthase 1 (soluble)	1.66 ↓	1.87 ↓
201742_x_at	*SRSF1*	serine/arginine-rich splicing factor 1	1.61 ↓	1.72 ↓
208290_s_at	*EIF5*	eukaryotic translation initiation factor 5	1.54 ↓	1.63 ↓
205529_s_at	*RUNX1T1*	runt-related transcription factor 1; translocated to, 1 (cyclin D-related)	1.53 ↑	1.6 ↑
202268_s_at	*NAE1*	NEDD8 activating enzyme E1 subunit 1	2.02 ↓	1.57 ↓
202232_s_at	*EIF3M*	eukaryotic translation initiation factor 3, subunit M	1.72 ↓	1.56 ↓

## Data Availability

The raw data supporting the conclusions of this article will be made available by the authors on request.

## References

[1] Wojciechowska U., Barańska K., Miklewska M., Didkowska J.A. (2023). Cancer incidence and mortality in Poland in 2020. J. Oncol..

[2] Gupta S., Kumar Y. (2022). Cancer prognosis using artificial intelligence-based techniques. SN Comput. Sci..

[3] Jenkins R.W., Fisher D.E. (2021). Treatment of advanced melanoma in 2020 and beyond. J. Invest Dermatol..

[4] Gutteridge J., Halliwell B. (2018). Mini-Review: Oxidative stress, redox stress or redox success?. Biochem. Biophys. Res. Commun..

[5] Kim T.Y., Kim E.N., Jeong G.S. (2024). Therapeutic effects of hinokitiol through regulating the SIRT1/NOX4 against ligature-induced experimental periodontitis. Antioxidants.

[6] Chen H.-Y., Cheng W.-P., Chiang Y.-F., Hong Y.-H., Ali M., Huang T.-C., Wang K.-L., Shieh T.-M., Chang H.-Y., Hsia S.-M. (2021). Hinokitiol Exhibits Antitumor Properties through Induction of ROS-Mediated Apoptosis and p53-Driven Cell-Cycle Arrest in Endometrial Cancer Cell Lines (Ishikawa, HEC-1A, KLE). Int. J. Mol. Sci..

[7] Shih Y.-H., Chang K.-W., Hsia S.-M., Yu C.-C., Fuh L.-J., Chi T.-Y., Shieh T.-M. (2013). In vitro antimicrobial and anticancer potential of hinokitiol against oral pathogens and oral cancer cell lines. Microbiol. Res..

[8] Ahmed F.H., Abdelgilil M.E., El-Sayed W.M. (2026). Hinokitiol as a Promising Anticancer Agent: Mechanisms of Action, Potential in Combination Therapy, and Overcoming Chemoresistance. Rev. Curr. Pharm. Des..

[9] Chiang Y.-F., Huang K.-C., Chen H.-Y., Hamdy N.M., Huang T.-C., Chang H.-Y., Shieh T.-M., Huang Y.-J., Hsia S.-M. (2024). Hinokitiol Inhibits Breast Cancer Cells In Vitro Stemness-Progression and Self-Renewal with Apoptosis and Autophagy Modulation via the CD44/Nanog/SOX2/Oct4 Pathway. Int. J. Mol. Sci..

[10] Seno S., Kimura M., Yashiro Y., Kimura R., Adachi K., Terabayashi A., Takahashi M., Oyama T., Abe H., Abe T. (2021). B-Thujaplicin Enhances TRAIL-Induced Apoptosis via the Dual Effects of XIAP Inhibition and Degradation in NCI-H460 Human Lung Cancer Cells. Medicines.

[11] Synowiec-Wojtarowicz A., Krawczyk A., Kimsa-Dudek M. (2024). Static Magnetic Field Reduces the Anticancer Effect of Hinokitiol on Melanoma Malignant Cells—Gene Expression and Redox Homeostasis Studies. Pharmaceuticals.

[12] Wang S., Zheng M., Lou C., Chen S., Guo H., Gao Y., Lv H., Yuan X., Zhang X., Shang P. (2021). Evaluating the biological safety on mice at 16 T static magnetic field with 700 MHz radio-frequency electromagnetic field. Ecotoxicol. Environ. Saf..

[13] Batistatou E., Molter A., Kromhout H., Van Tongeren M., Crozier S., Schaap K., Gowland P., Keevil S.F., De Vocht F. (2016). Personal exposure to static and time-varying magnetic fields during MRI procedures in clinical practice in the UK. Occup. Environ. Med..

[14] Tian X., Lv Y., Fan Y., Wang Z., Yu B., Song C., Lu Q., Xi C., Li P., Zhang X. (2021). Safety evaluation of mice exposed to 7.0–33.0 T high-static magnetic fields. J. Magn. Reson. Imag..

[15] Albuquerque W.W., Costa R.M., Fernandes S., Porto A.L. (2016). Evidences of the static magnetic field influence on cellular systems. Prog. Biophys. Mol. Biol..

[16] Yang J., Feng Y., Li Q., Zeng Y. (2023). Evidence of the static magnetic field effects on bone-related diseases and bone cells. Prog. Biophys. Mol. Biol..

[17] Wang Z., Hao F., Ding C., Yang Z., Shang P. (2014). Effects of static magnetic field on cell biomechanical property and membrane ultrastructure. Bioelectromagnetics.

[18] Gurhan H., Bruzon R., Kandala S., Greenebaum B., Barnes F. (2021). Effects induced by a weak static magnetic field of different intensities on HT-1080 fibrosarcoma cells. Bioelectromagnetics.

[19] Song C., Yu B., Wang J., Ji X., Zhang L., Tian X., Yu X., Feng C., Wang X., Zhang X. (2021). Moderate static magnet fields suppress ovarian cancer metastasis via ROS-mediated oxidative stress. Oxid. Med. Cell Longev..

[20] Bolick N.L., Geller A.C. (2024). Epidemiology and Screening for Melanoma. Hematol. Oncol. Clin. N. Am..

[21] Leiter U., Keim U., Garbe C. (2020). Epidemiology of skin cancer: Update 2019. Adv. Exp. Med. Biol..

[22] Hu S., Chu Y., Zhou X., Wang X. (2023). Recent advances of ferroptosis in tumor: From biological function to clinical application. BioMed. Pharmacother..

[23] Ciu X., Dong X., Li Y., Yuan Z., Wang L., Zhang L., Ma Y. (2026). Harnessing autophagy-dependent ferroptosis: Mechanisms and therapeutic opportunities in human diseases. Pharmacol. Res..

[24] Kong D., Liu Y., Zhang M. (2022). Expression of the Circadian Clock Gene ARNTL associated with DNA repair gene and prognosis of patient with osteosarcoma. Mut. Res.-Fund. Mol. Mech. Mutagen..

[25] Jiang W., Zhao S., Shen J., Guo L., Sun Y., Zgu Y., Ma Z., Zhang X., Hu Y., Xiao W. (2018). The MiR-135b–BMAL1–YY1 loop disturbs pancreatic clockwork to promote tumourigenesis and chemoresistance. Cell Death Dis..

[26] Zeng Z.L., Luo H.Y., Yang J., Wu W.J., Chen D.L., Huang P., Xu R. (2014). Overexpression of the circadian clock gene Bmal1 increases sensitivity to oxaliplatin in colorectal cancer. Clin. Cancer Res. Off. J. Am. Assoc. Cancer Res..

[27] Zhu J., Zhou Q., Pan M., Zhou C. (2021). Multi-omics analysis of the prognosis and therapeutic significance of circadian clock in ovarian cancer. Gene.

[28] Zhang J.F., Liu J., Wu J.L., Li W.F., Chen Z.W., Yang L.S. (2019). Progression of the role of CRYAB in signaling pathways and cancers. Onco Targets Ther..

[29] Qiao S., Lamore S.D., Cabello C.H.M., Lesson J.L., Munoz-Rodriguez J.L., Wondrak G.T. (2012). Thiostrepton is an inducer of oxidative and proteotoxic stress that impairs viability of human melanoma cells but not primary melanocytes. Biochem Pharmacol..

[30] Tan L., Sha L., Hou N., Zhang M., Ma Q., Shi C. (2019). High α B-crystallin and p53 co-expression is associated with poor prognosis in ovarian cancer. Biosci. Rep..

[31] Jayakumar M.N., Muhammad J.S., Dutta M., Donakonda S. (2023). Comprehensive In silico analysis of chaperones identifies CRYAB and P4HA2 as potential therapeutic targets and their small-molecule inhibitors for the treatment of cholangiocarcinoma. Comput. Biol. Med..

[32] Zhang Y.-Y., Zeng Q.-X., Wang L., Li K.-X., Zhang S., Wan P., Zhou X.-M., Chen Q. (2025). Norcantharidin inhibits the METTL16/MAT2A pathway to induce apoptosis and suppress tumor progression in ovarian cancer. Arch. Biochem. Biophys..

[33] Li C., Lan X., Li X., Fu Y., Gui G., Li X., Shen Y., Gan Z., Huang M. (2025). Discovery of 2(1H)-Quinoxalinone Derivatives as Potent and Selective MAT2A Inhibitors for the Treatment of MTAP-Deficient Cancers. J. Med. Chem..

[34] Qin M., Shao B., Lin L., Zhang Z.-Q., Sheng Z.-G., Qin L., Shao J., Zhu B.-Z. (2023). Molecular mechanism of the unusual biphasic effects of the natural compound hinokitiol on iron-induced cellular DNA damage. Free. Radic. Biol. Med..

[35] Lu S., Song Y., Luo R., Li S., Li G., Wang K., Liao Z., Wang B., Ke W., Xiang Q. (2021). Ferroportin-dependent iron homeostasis protects against oxidative stress-induced nucleus pulposus cell ferroptosis and ameliorates intervertebral disc degeneration in vivo. Oxidative Med. Cell. Longev..

[36] Zhang X., Zhang M., Zhang Z., Zhou S. (2024). Salidroside induces mitochondrial dysfunction and ferroptosis to inhibit melanoma progression through reactive oxygen species production. Exp. Cell Res..

[37] Liu T., Liu H., Wang P., Hu Y., Yang R., Liu F., Kim H.G., Dong Z., Liu K. (2020). Honokiol Inhibits Melanoma Growth by Targeting Keratin 18 in vitro and in vivo. Front Cell Dev. Biol..

[38] Hu X., Li J., Yu L., Ifejola J., Guo Y., Zhang D., Khosravi Z., Zhang K., Cui H. (2024). Screening of anti-melanoma compounds from Morus alba L.: Sanggenon C promotes melanoma cell apoptosis by disrupting intracellular Ca2+ homeostasis. J. Ethnopharmacol..

[39] Glinka M., Gawron S., Sieroń A., Pawłowska-Góral K., Cieślar G., Sieroń-Stołtny K. (2013). Test chambers for cell culture in static magnetic field. J. Magn. Magn. Mat..

[40] Kimsa-Dudek M., Synowiec-Wojtarowicz A., Derewniuk M., Gawron S., Paul-Samojedny M., Kruszniewska-Rajs C., Pawłowska-Góral K. (2018). Impact of fluoride and a static magnetic field on the gene expression that is associated with the antioxidant defense system of human fibroblasts. Chem. Biol. Interact..

